# The genome sequence of the common water plantain, *Alisma plantago-aquatica* L. (Alismataceae)

**DOI:** 10.12688/wellcomeopenres.24005.1

**Published:** 2025-04-23

**Authors:** Maarten J. M. Christenhusz, Sahr Mian, Ilia J. Leitch, Royal Botanic Gardens Kew Genome Acquisition Lab, Darwin Tree of Life Barcoding collective, Plant Genome Sizing collective, Wellcome Sanger Institute Tree of Life Management, Samples and Laboratory team, Wellcome Sanger Institute Scientific Operations: Sequencing Operations, Wellcome Sanger Institute Tree of Life Core Informatics team, Tree of Life Core Informatics collective, Darwin Tree of Life Consortium

**Affiliations:** 1 Royal Botanic Gardens Kew, Richmond, England, UK; 2 Curtin University, Perth, Western Australia, Australia

**Keywords:** Alisma plantago-aquatica, common water plantain, genome sequence, chromosomal, Alismatales

## Abstract

We present a genome assembly from a specimen of *Alisma plantago-aquatica* (common water plantain; Streptophyta; Magnoliopsida; Alismatales; Alismataceae). The genome sequence has a total length of 9,377.97 megabases. Most of the assembly (99.53%) is scaffolded into 7 chromosomal pseudomolecules. The mitochondrial and plastid genome assemblies have lengths of 250.4 kilobases and 159.88 kilobases, respectively.

## Species taxonomy

Eukaryota; Viridiplantae; Streptophyta; Streptophytina; Embryophyta; Tracheophyta; Euphyllophyta; Spermatophyta; Magnoliopsida; Mesangiospermae; Liliopsida; Alismatales; Alismataceae; *Alisma*; *Alisma plantago-aquatica* L. (NCBI:txid15000).

## Background

**Figure 1.**
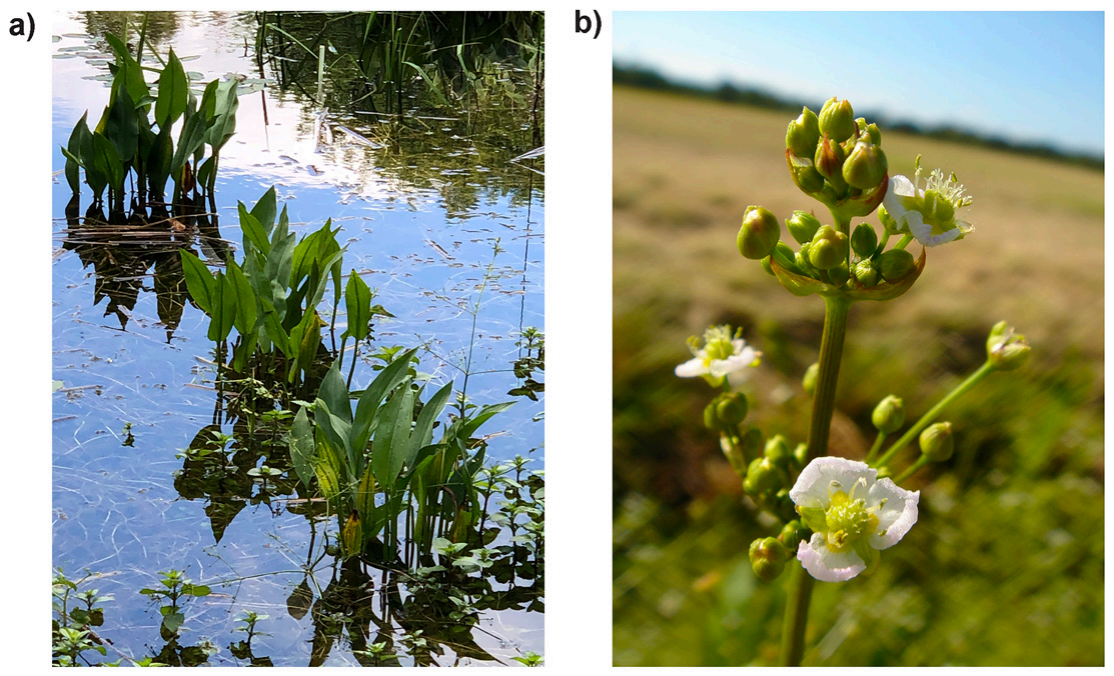
Photographs of *Alisma plantago-aquatica* **a**) Photograph of the population in Wytham Woods from which the sequenced specimen was sampled, **b**) Flowers from a plant in Ruissalo, Finland. Photographs by Maarten Christenhusz.

Common water plantain, *Alisma plantago-aquatica* L., is a perennial, emergent aquatic herb ( Figure 1). It grows in wet soil, such as exposed mud or on the edge of shallow, slow-flowing or still water bodies, such as lakes, streams, ditches, canals, marshes or swamps that are often relatively nutrient-rich. The species is typically confined to lowland habitats, but has been recorded at 460 m in Shropshire ( Stroh *et al*., 2020). It is native and widespread across the UK as well as across Eurasia, extending into North and East Africa. It is also widely naturalised in southern South America, southern Africa, Australia and New Zealand ( POWO, 2025).

It has broad-lanceolate, undivided leaves that emerge from of the water surface, and an inflorescence with whorled branches, bearing numerous flowers. Flowers have three small green sepals and three rounded tepals that are white or tinged light purple/pink and that often have a jagged edge. The six stamens surround the numerous free carpels that after pollination form small beaked achenes. Plants make copious seed and can be found colonising new aquatic habitats, such as new ponds, new water edges and fresh-water mudflats. Studies on the pollination biology of *A. plantago-aquatica* suggest that while the species may be self-compatible, cross-pollination by insects is most efficient, with hoverflies and bees being the most common pollinators ( Vislobokov & Kuzmicheva, 2022).

The venation of the leaves of *Alisma* inspired British art and social critic John Ruskin (1819–1900). In his eyes the curvature represented divine proportion, as seen in many Gothic arches, influencing his theories of Gothic architecture ( Mordaunt Crook, 1982; Rice *et al.*, 2015).

Chromosome studies have shown that the species is diploid, with 2 *n* = 2 *x* = 14 consistently reported ( Montgomery *et al.*, 1997) across the range of the species. This is the first species to be sequenced to chromosome-level for Alismataceae and will be an important resource to improve investigations on the biosynthetic pathways for some of the compounds that may be of medicinal value. It may also help resolve some taxonomic issues surrounding related species and explore if there is any evidence of possible hybridisation in the genus. The genome of *Alisma plantago-aquatica* was sequenced as part of the Darwin Tree of Life Project, based on a specimen sampled from Wytham Woods, Oxfordshire, UK.

## Genome sequence report

### Sequencing data

The genome of a specimen of *Alisma plantago-aquatica* was sequenced using Pacific Biosciences single-molecule HiFi long reads, generating 243.05 Gb (gigabases) from 20.82 million reads. GenomeScope analysis of the PacBio HiFi data estimated the haploid genome size at 9,558.92 Mb, with a heterozygosity of 0.11% and repeat content of 50.31%. These values provide an initial assessment of genome complexity and the challenges anticipated during assembly. Based on this estimated genome size, the sequencing data provided approximately 27.0x coverage of the genome. Using flow cytometry, the genome size (1C-value) was estimated to be 10.81 pg, equivalent to 10,580 Mb. Primary assembly contigs were scaffolded with chromosome conformation Hi-C data, which produced 265.28 Gb from 1,756.79 million reads. Table 1 summarises the specimen and sequencing information.

**Table 1.** Specimen and sequencing data for *Alisma plantago-aquatica*.

Project information			
**Study title**	Alisma plantago-aquatica (common water plantain)		
**Umbrella BioProject**	PRJEB65656		
**Species**	*Alisma plantago-aquatica*		
**BioSample**	SAMEA9335272		
**NCBI taxonomy ID**	15000		
Specimen information			
**Technology**	**ToLID**	**BioSample accession**	**Organism part**
**PacBio long read sequencing**	laAliPlan1	SAMEA9335416	leaf
**Hi-C sequencing**	laAliPlan1	SAMEA9335416	leaf
**RNA sequencing**	laAliPlan1	SAMEA9335416	leaf
Sequencing information			
**Platform**	**Run accession**	**Read count**	**Base count (Gb)**
**Hi-C Illumina NovaSeq 6000**	ERR12035225	6.41e+09	967.86
**Hi-C Illumina NovaSeq 6000**	ERR12035226	1.76e+09	265.28
**PacBio Sequel IIe**	ERR12015725	1.87e+06	18.85
**PacBio Revio**	ERR12015723	4.33e+06	53.26
**PacBio Revio**	ERR12015724	4.70e+06	56.28
**PacBio Sequel IIe**	ERR12015720	2.17e+06	24.92
**PacBio Sequel IIe**	ERR12015721	0.00e+00	0.0
**PacBio Revio**	ERR12015722	5.62e+06	68.4
**PacBio Sequel IIe**	ERR12015726	2.14e+06	21.33
**RNA Illumina NovaSeq 6000**	ERR12035227	6.33e+07	9.56

### Assembly statistics

The primary haplotype was assembled, and contigs corresponding to an alternate haplotype were also deposited in INSDC databases. Manual assembly curation corrected six missing joins or mis-joins. The final assembly has a total length of 9,377.97 Mb in 236 scaffolds, with 249 gaps, and a scaffold N50 of 1500.0 Mb ( Table 2).

**Table 2.** Genome assembly data for *Alisma plantago-aquatica*, laAliPlan1.1.

Genome assembly		
Assembly name	laAliPlan1.1	
Assembly accession	GCA_963693085.1	
*Accession of alternate haplotype*	*GCA_963693085.1*	
Span (Mb)	9,378.00	
Number of contigs	487	
Number of scaffolds	236	
Longest scaffold (Mb)	1,999.79	
Assembly metrics for the primary assembly		*Benchmark*
Contig N50 length (Mb)	87.7	*≥ 1 Mb*
Scaffold N50 length (Mb)	1,500.0	*= chromosome N50*
Consensus quality (QV)	Primary: 65.5; alternate: 53.2; combined 64.4	*≥ 40*
*k*-mer completeness	Primary: 99.53%; alternate: 2.33%; combined: 99.54%	*≥ 95%*
BUSCO *	C:85.5%[S:80.4%,D:5.0%], F:8.2%,M:6.3%,n:3,236	*S > 90%; D < 5%*
Percentage of assembly mapped to chromosomes	99.53%	*≥ 90%*
Organelles	Mitochondrial genome: 250.4 kb; Plastid genome: 159.88 kb	*complete single alleles*

* BUSCO scores based on the liliopsida_odb10 BUSCO set using version 5.5.0. C = complete [S = single copy, D = duplicated], F = fragmented, M = missing, n = number of orthologues in comparison. A full set of BUSCO scores is available at https://blobtoolkit.genomehubs.org/view/Alisma_plantago-aquatica/dataset/GCA_963693085.1/busco.

**Figure 2.**
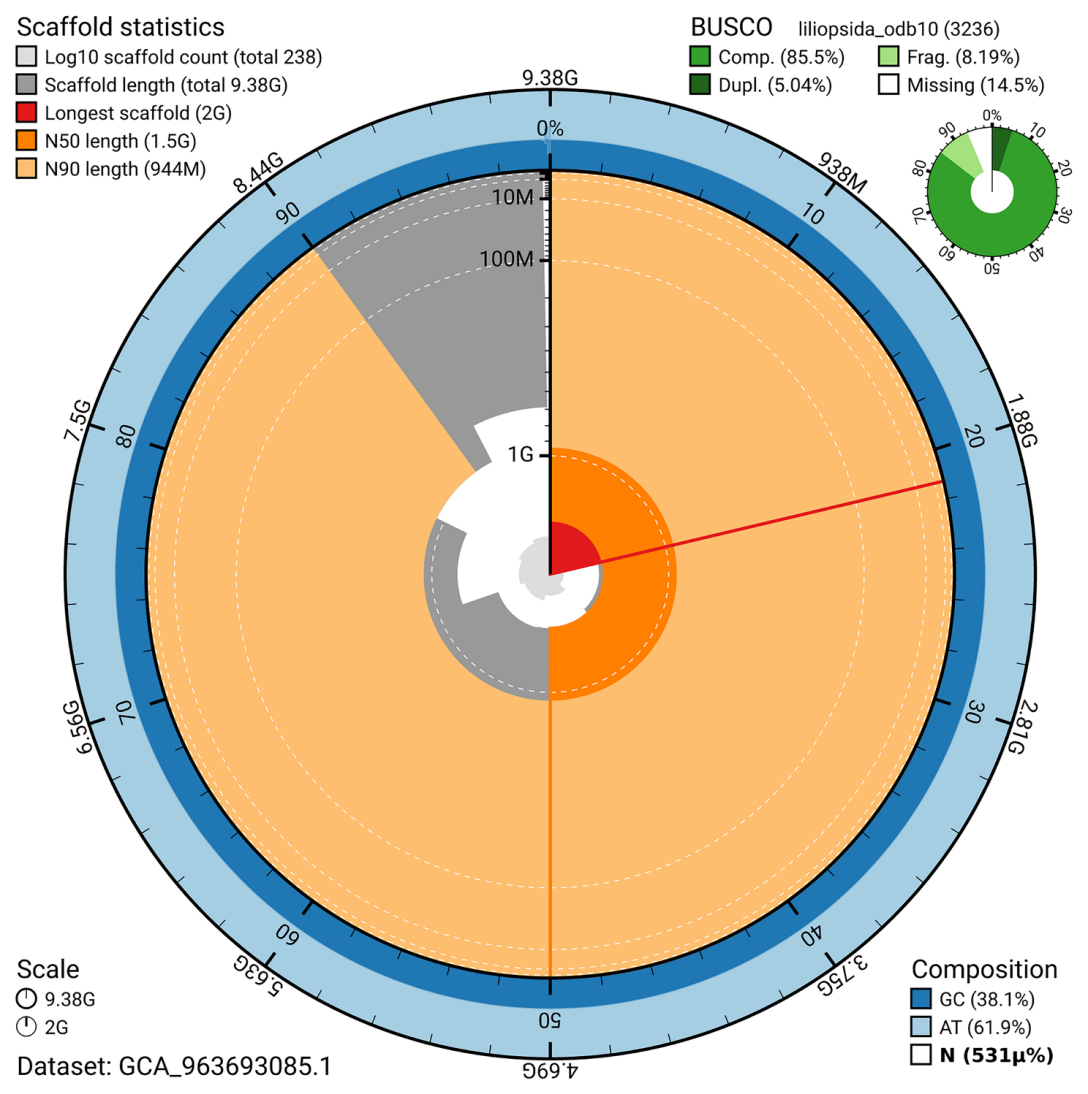
Genome assembly of *Alisma plantago-aquatica*, laAliPlan1.1: metrics. The BlobToolKit snail plot shows N50 metrics and BUSCO gene completeness. The main plot is divided into 1,000 bins around the circumference with each bin representing 0.1% of the 9,378,379,957 bp assembly. The distribution of scaffold lengths is shown in dark grey with the plot radius scaled to the longest scaffold present in the assembly (1,999,785,258 bp, shown in red). Orange and pale-orange arcs show the N50 and N90 scaffold lengths (1,499,997,841 and 943,684,407 bp), respectively. The pale grey spiral shows the cumulative scaffold count on a log scale with white scale lines showing successive orders of magnitude. The blue and pale-blue area around the outside of the plot shows the distribution of GC, AT and N percentages in the same bins as the inner plot. A summary of complete, fragmented, duplicated and missing BUSCO genes in the liliopsida_odb10 set is shown in the top right. An interactive version of this figure is available at https://blobtoolkit.genomehubs.org/view/GCA_963693085.1/dataset/GCA_963693085.1/snail.

**Figure 3.**
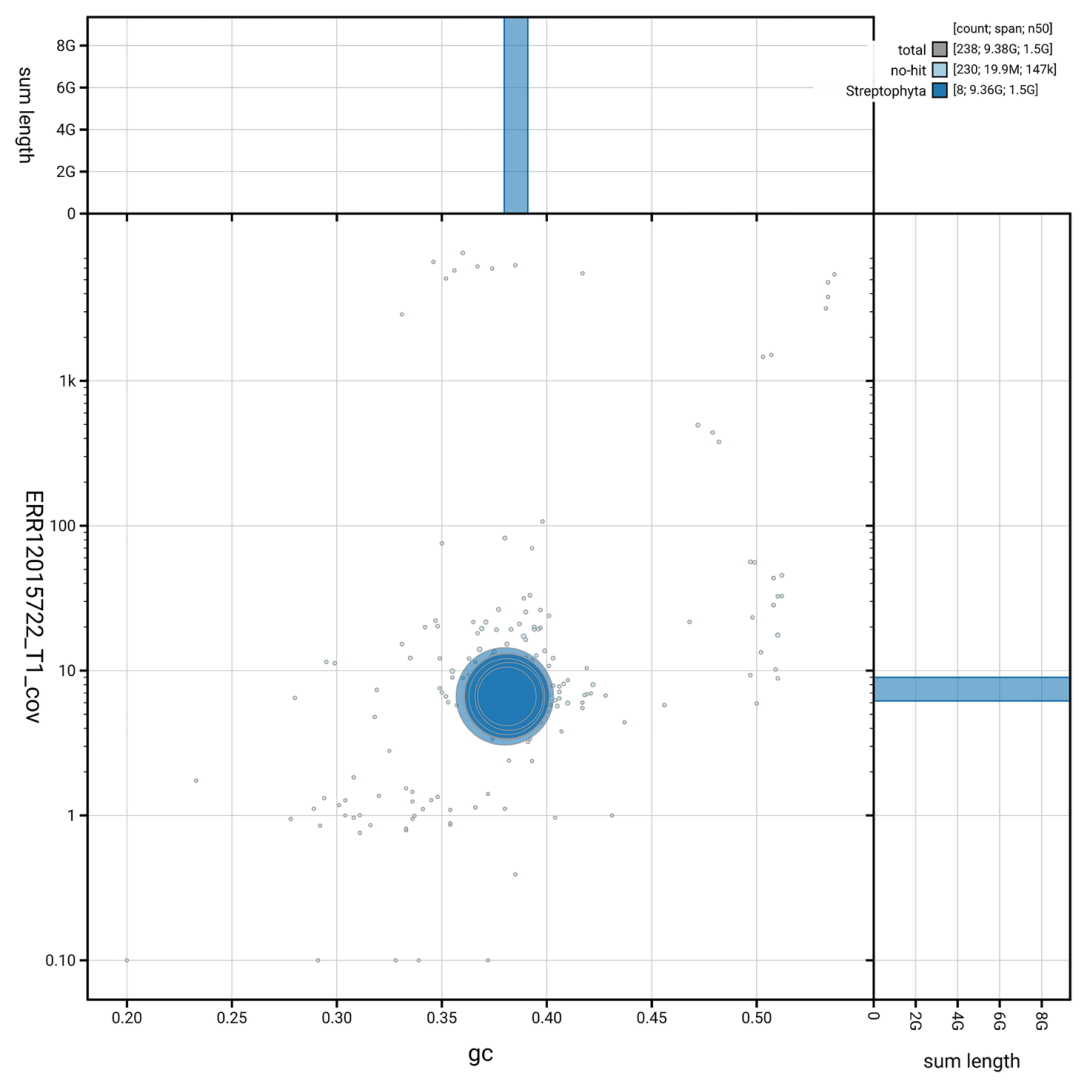
Genome assembly of *Alisma plantago-aquatica*, laAliPlan1.1: BlobToolKit GC-coverage plot. Scaffolds are coloured by phylum. Circles are sized in proportion to scaffold length. Histograms show the distribution of scaffold length sum along each axis. An interactive version of this figure is available at https://blobtoolkit.genomehubs.org/view/GCA_963693085.1/dataset/GCA_963693085.1/blob.

**Figure 4.**
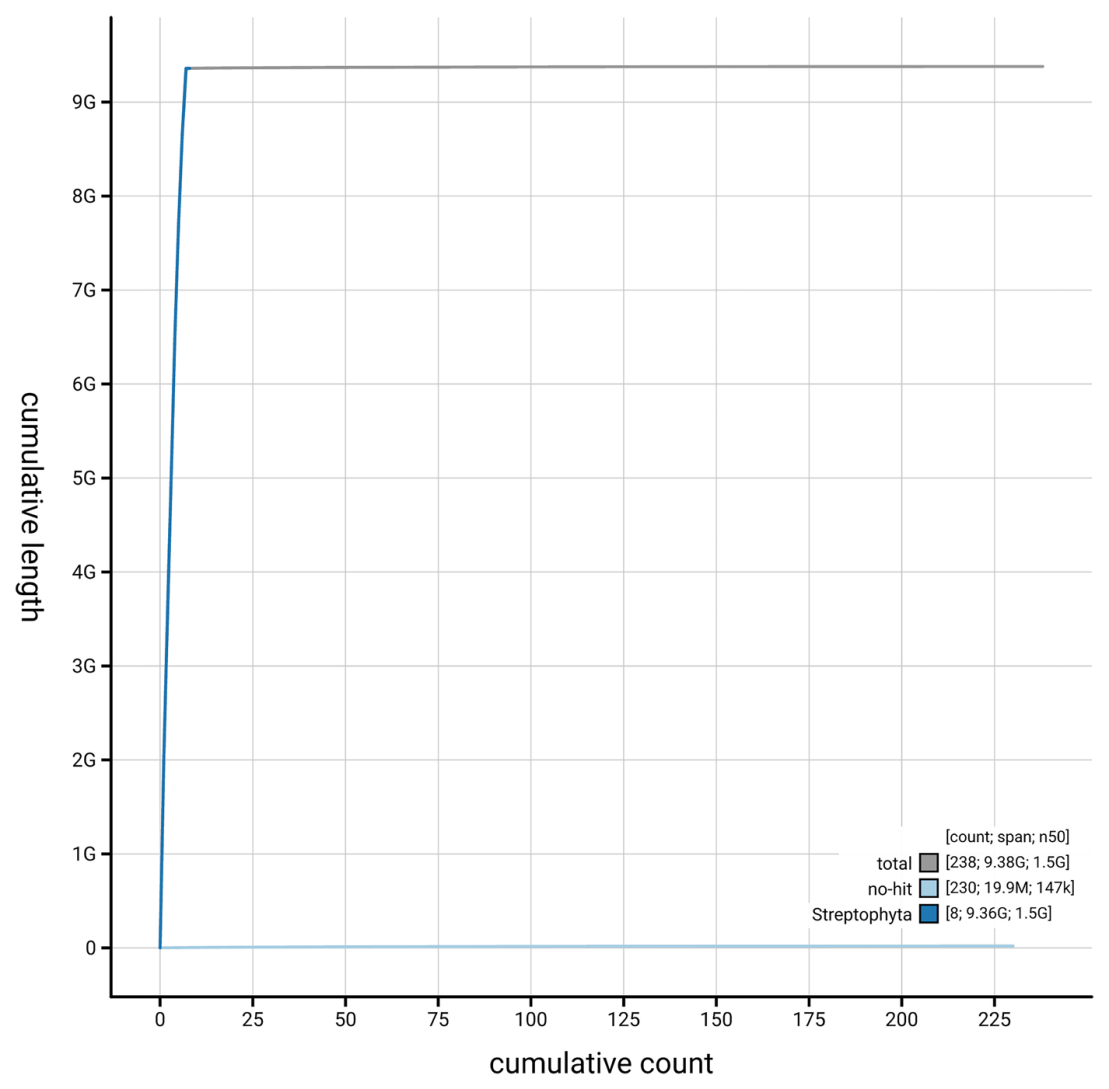
Genome assembly of *Alisma plantago-aquatica*, laAliPlan1.1: BlobToolKit cumulative sequence plot. The grey line shows cumulative length for all scaffolds. Coloured lines show cumulative lengths of scaffolds assigned to each phylum using the buscogenes taxrule. An interactive version of this figure is available at https://blobtoolkit.genomehubs.org/view/GCA_963693085.1/dataset/GCA_963693085.1/cumulative.

**Figure 5.**
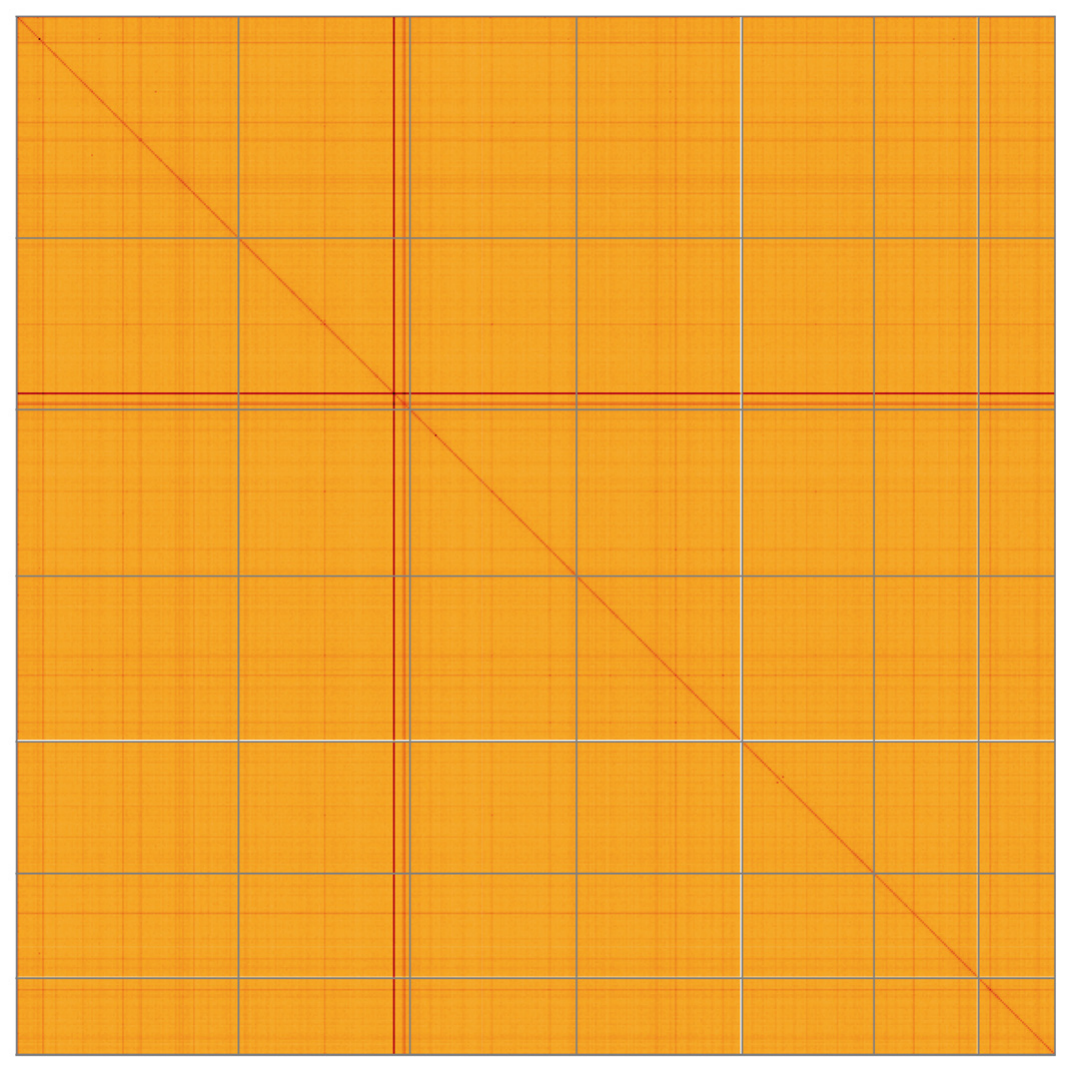
Genome assembly of *Alisma plantago-aquatica*, laAliPlan1.1: Hi-C contact map of the laAliPlan1.1 assembly, visualised using HiGlass. Chromosomes are shown in order of size from left to right and top to bottom. An interactive version of this figure may be viewed at https://genome-note-higlass.tol.sanger.ac.uk/l/?d=f6QPEY1CSTytufDMVmezGg.

The snail plot in Figure 2 provides a summary of the assembly statistics, while the distribution of assembly scaffolds by GC proportion and coverage is shown in Figure 3. The cumulative assembly plot in Figure 4 shows curves for subsets of scaffolds assigned to different phyla. Most (99.78%) of the assembly sequence was assigned to 7 chromosomal-level scaffolds. Chromosome-scale scaffolds confirmed by the Hi-C data are named in order of size ( Figure 5; Table 3). The mitochondrial and plastid genomes were also assembled and can be found as contigs within the multifasta file of the genome submission.

**Table 3.** Chromosomal pseudomolecules in the genome assembly of *Alisma plantago-aquatica*, laAliPlan1.

INSDC accession	Name	Length (Mb)	GC%
OY856409.1	1	1999.79	38
OY856410.1	2	1545.73	38
OY856411.1	3	1500.0	38
OY856412.1	4	1493.21	38
OY856413.1	5	1187.61	38
OY856414.1	6	943.68	38
OY856415.1	7	688.36	38
OY856416.1	MT	0.25	47
OY856417.1	Pltd	0.16	36

### Assembly quality metrics

The estimated Quality Value (QV) and *k*-mer completeness metrics, along with BUSCO completeness scores, were calculated for each haplotype and the combined assembly. The QV reflects the base-level accuracy of the assembly, while *k*-mer completeness indicates the proportion of expected *k*-mers identified in the assembly. BUSCO scores provide a measure of completeness based on benchmarking universal single-copy orthologues.

The primary haplotype has a QV of 65.5, and the combined primary and alternate assemblies achieve an estimated QV of 64.4. The *k*-mer recovery for the primary haplotype is 99.53%, and for the alternate haplotype 2.33%; the combined primary and alternate assemblies have a *k*-mer recovery of 99.54%. BUSCO v.5.5.0 analysis using the liliopsida_odb10 reference set ( *n* = 3,236) identified 85.5% of the expected gene set (single = 80.4%, duplicated = 5.0%).

Table 2 provides assembly metric benchmarks adapted from Rhie *et al.* (2021) and the Earth BioGenome Project (EBP) Report on Assembly Standards September 2024. The assembly achieves the EBP reference standard of **7.C.Q65**.

## Methods

### Sample acquisition, DNA barcoding and genome size estimation

A specimen of *Alisma plantago-aquatica* (specimen ID KDTOL10264, ToLID laAliPlan1) was picked by hand in Wytham Woods, Oxfordshire, UK (latitude 51.76, longitude –1.34) on 2021-06-22. The specimen was collected by Maarten Christenhusz, Sahr Mian and Ilia Leitch (Royal Botanic Gardens Kew; RBG Kew; collection number MC9202), identified by Maarten Christenhusz, and preserved in the field by freezing at –80 °C. The herbarium voucher associated with the sequenced plant was deposited in the herbarium of RBG Kew (K).

The genome size was estimated by flow cytometry at RBG Kew using the fluorochrome propidium iodide and following the ‘one-step’ method as outlined in Pellicer *et al.* (2021). For this species, CyStain™ PI OxProtect Staining Buffer (cat. No. 05-5027; Sysmex UK Ltd.) was used for isolation of nuclei ( Loureiro *et al.*, 2007), and the internal calibration standard was *Pisum sativum* ‘Ctirad’ with an assumed 1C-value of 4,445 Mb ( Doležel *et al.*, 1998).

The initial species identification was verified by an additional DNA barcoding process following the framework developed by Twyford *et al*. (2024). Part of the plant specimen was preserved in silica gel desiccant ( Chase & Hills, 1991). DNA was extracted from the dried specimen, then PCR was used to amplify standard barcode regions. The resulting amplicons were sequenced and compared to public sequence databases including GenBank and the Barcode of Life Database (BOLD). The barcode sequences for this specimen are available on BOLD ( Ratnasingham & Hebert, 2007). Following whole genome sequence generation, DNA barcodes were also used alongside the initial barcoding data for sample tracking through the genome production pipeline at the Wellcome Sanger Institute (WSI; Twyford *et al.*, 2024). The standard operating procedures for the Darwin Tree of Life barcoding have been deposited on protocols.io ( Beasley *et al.*, 2023).

### Nucleic acid extraction

The workflow for high molecular weight (HMW) DNA extraction at the WSI Tree of Life Core Laboratory includes a sequence of core procedures: sample preparation and homogenisation, DNA extraction, fragmentation and purification. Detailed protocols are available on protocols.io ( Denton *et al.*, 2023). The laAliPlan1 sample was weighed and dissected on dry ice ( Jay *et al.*, 2023) and leaf tissue was cryogenically disrupted using the Covaris cryoPREP ^®^ Automated Dry Pulverizer ( Narváez-Gómez *et al.*, 2023).

HMW DNA was extracted using the Plant Organic DNA Extraction Protocol ( Jackson & Howard, 2023). HMW DNA was sheared into an average fragment size of 12–20 kb in a Megaruptor 3 system ( Todorovic *et al.*, 2023). Sheared DNA was purified by solid-phase reversible immobilisation ( Strickland *et al.*, 2023). The concentration of the sheared and purified DNA was assessed using a Nanodrop spectrophotometer and Qubit Fluorometer using a Qubit dsDNA High Sensitivity Assay kit. Fragment size distribution was evaluated by running the sample on the FemtoPulse system.

RNA was extracted from leaf tissue of laAliPlan1 in the Tree of Life Laboratory at the WSI using the RNA Extraction: Automated MagMax™ *mir*Vana protocol ( do Amaral *et al.*, 2023). The RNA concentration was assessed using a Nanodrop spectrophotometer and a Qubit Fluorometer using the Qubit RNA Broad-Range Assay kit. Analysis of the integrity of the RNA was done using the Agilent RNA 6000 Pico Kit and Eukaryotic Total RNA assay.

### Hi-C sample preparation

Hi-C data were generated from the leaf tissue of laAliPlan1 using the Arima-HiC v2 kit at the WSI Scientific Operations core. Tissue was finely ground using cryoPREP and then subjected to nuclei isolation. Nuclei were isolated using a modified protocol of the Qiagen QProteome Cell Compartment Kit where only CE1 and CE2 buffers are used in combination with QiaShredder spin columns. After isolation, the nuclei were fixed using 37% formaldehyde solution to crosslink the DNA. The crosslinked DNA was then digested using the restriction enzyme master mix. The 5’-overhangs were then filled in and labelled with biotinylated nucleotides and proximally ligated. An overnight incubation was carried out for enzymes to digest remaining proteins and for crosslinks to reverse. A clean up was performed with SPRIselect beads prior to library preparation. DNA concentration was quantified using the Qubit Fluorometer v4.0 and Qubit HS Assay Kit according to the manufacturer’s instructions.

### Library preparation and sequencing

Library preparation and sequencing were performed at the WSI Scientific Operations core.

***PacBio HiFi***

At a minimum, samples were required to have an average fragment size exceeding 8 kb and a total mass over 400 ng to proceed to the low input SMRTbell Prep Kit 3.0 protocol (Pacific Biosciences, California, USA). Libraries were prepared using the SMRTbell Prep Kit 3.0 (Pacific Biosciences, California, USA) as per the manufacturer’s instructions. The kit includes the reagents required for end repair/A-tailing, adapter ligation, post-ligation SMRTbell bead cleanup, and nuclease treatment. Following the manufacturer’s instructions, size selection and clean up was carried out using diluted AMPure PB beads (Pacific Biosciences, California, USA). DNA concentration was quantified using the Qubit Fluorometer v4.0 (Thermo Fisher Scientific) with Qubit 1X dsDNA HS assay kit and the final library fragment size analysis was carried out using the Agilent Femto Pulse Automated Pulsed Field CE Instrument (Agilent Technologies) and gDNA 55kb BAC analysis kit.

Samples were sequenced on a Sequel IIe instrument (Pacific Biosciences, California, USA). The concentration of the library loaded onto the Sequel IIe was in the range 40–135 pM. The SMRT link software, a PacBio web-based end-to-end workflow manager, was used to set-up and monitor the run, as well as perform primary and secondary analysis of the data upon completion.

Sequencing was also performed on a Revio instrument (Pacific Biosciences, California, USA). Prepared libraries were normalised to 2 nM, and 15 μL was used for making complexes. Primers were annealed and polymerases were hybridised to create circularised complexes, according to manufacturer’s instructions. The complexes were purified with the 1.2X clean up with SMRTbell beads. The purified complexes were then diluted to the Revio loading concentration, in the range 200–300 pM, and spiked with a Revio sequencing internal control. Samples were sequenced on Revio 25M SMRT cells. The SMRT link software, a PacBio web-based end-to-end workflow manager, was used to set-up and monitor the run, as well as perform primary and secondary analysis of the data upon completion.

***Hi-C***

For Hi-C library preparation, biotinylated DNA constructs were fragmented using the Covaris E220 sonicator (Covaris) and size selected using SPRISelect beads to 400 to 600 bp using the INTEGRA MINI 96 (INTEGRA). The DNA was then enriched using the Arima-HiC v2 kit Enrichment beads on the INTEGRA MINI 96 (INTEGRA). The NEBNext Ultra II DNA Library Prep Kit (New England Biolabs) was used for end repair, A-tailing, and adapter ligation. This uses a custom protocol which resembles the standard NEBNext Ultra II DNA Library Prep protocol but where library preparation occurs while DNA is bound to the Enrichment beads. Library PCR amplification was carried out using KAPA HiFi Hot start mix and a custom IDT UDI (Unique Dual Index) 96 barcode plate (Integrated DNA Technologies). Depending on sample concentration and biotinylation percentage determined at the crosslinking stage, samples were run for 10–16 PCR cycles. Post-PCR, samples were cleaned-up using SPRISelect beads and the INTEGRA MINI 96 (INTEGRA). The libraries were quantified using the Accuclear Ultra High Sensitivity dsDNA Standards Assay kit (Biotium), Mosquito LV liquid handling platform (SPT Labtech), Agilent Bravo Work Station (Agilent) and BMG FLUOstar Omega plate reader (BMG Labtech). Hi-C sequencing was performed using paired-end sequencing with a read length of 150 bp on an Illumina NovaSeq 6000 instrument.

***RNA***

Poly(A) RNA-Seq libraries were constructed using the NEB Ultra II RNA Library Prep kit, following the manufacturer’s instructions. RNA sequencing was performed on the Illumina NovaSeq 6000 instrument.

### Genome assembly, curation and evaluation

***Assembly***

The HiFi reads were first assembled using Hifiasm ( Cheng *et al.*, 2021) with the --primary option. Haplotypic duplications were identified and removed with purge_dups ( Guan *et al.*, 2020). Hi-C reads were further mapped with bwa-mem2 ( Vasimuddin *et al.*, 2019) to the primary contigs, which were further scaffolded using the provided Hi-C data ( Rao *et al.*, 2014) in YaHS ( Zhou *et al.*, 2023) using the --break option. Scaffolded assemblies were evaluated using Gfastats ( Formenti *et al.*, 2022), BUSCO ( Manni *et al.*, 2021) and MERQURY.FK ( Rhie *et al.*, 2020). The organelle genomes were assembled using OATK ( Zhou, 2023).

***Curation***

The assembly was decontaminated using the Assembly Screen for Cobionts and Contaminants (ASCC) pipeline (article in preparation). Flat files and maps used in curation were generated in TreeVal ( Pointon *et al.*, 2023). Manual curation was primarily conducted using PretextView ( Harry, 2022), with additional insights provided by JBrowse2 ( Diesh *et al.*, 2023) and HiGlass ( Kerpedjiev *et al.*, 2018). Scaffolds were visually inspected and corrected as described by Howe *et al*. (2021). Any identified contamination, missed joins, and mis-joins were corrected, and duplicate sequences were tagged and removed. The process is documented at https://gitlab.com/wtsi-grit/rapid-curation (article in preparation).

***Evaluation of assembly quality***

The Merqury.FK tool ( Rhie *et al.*, 2020), run in a Singularity container ( Kurtzer *et al.*, 2017), was used to evaluate *k*-mer completeness and assembly quality for the primary and alternate haplotypes using the *k*-mer databases ( *k* = 31) computed prior to genome assembly. The analysis outputs included assembly QV scores and completeness statistics.

A Hi-C contact map was produced for the final version of the assembly. The Hi-C reads were aligned using bwa-mem2 ( Vasimuddin *et al.*, 2019) and the alignment files were combined using SAMtools ( Danecek *et al.*, 2021). The Hi-C alignments were converted into a contact map using BEDTools ( Quinlan & Hall, 2010) and the Cooler tool suite ( Abdennur & Mirny, 2020). The contact map was visualised in HiGlass ( Kerpedjiev *et al.*, 2018).

The blobtoolkit pipeline is a Nextflow port of the previous Snakemake Blobtoolkit pipeline ( Challis *et al.*, 2020). It aligns the PacBio reads in SAMtools and minimap2 ( Li, 2018) and generates coverage tracks for regions of fixed size. In parallel, it queries the GoaT database ( Challis *et al.*, 2023) to identify all matching BUSCO lineages to run BUSCO ( Manni *et al.*, 2021). For the three domain-level BUSCO lineages, the pipeline aligns the BUSCO genes to the UniProt Reference Proteomes database ( Bateman *et al.*, 2023) with DIAMOND ( Buchfink *et al.*, 2021) blastp. The genome is also split into chunks according to the density of the BUSCO genes from the closest taxonomic lineage, and each chunk is aligned to the UniProt Reference Proteomes database with DIAMOND blastx. Genome sequences with no hits are chunked with seqtk and aligned to the NT database with blastn ( Altschul *et al.*, 1990). The blobtools suite combines all these outputs into a blobdir for visualisation.

The blobtoolkit pipeline was developed using nf-core tooling ( Ewels *et al.*, 2020) and MultiQC ( Ewels *et al.*, 2016), relying on the Conda package manager, the Bioconda initiative ( Grüning *et al.*, 2018), the Biocontainers infrastructure ( da Veiga Leprevost *et al.*, 2017), as well as the Docker ( Merkel, 2014) and Singularity ( Kurtzer *et al.*, 2017) containerisation solutions.

Table 4 contains a list of relevant software tool versions and sources.

**Table 4.** Software tools: versions and sources.

Software tool	Version	Source
BEDTools	2.30.0	https://github.com/arq5x/bedtools2
BLAST	2.14.0	ftp://ftp.ncbi.nlm.nih.gov/blast/executables/blast+/
BlobToolKit	4.3.9	https://github.com/blobtoolkit/blobtoolkit
BUSCO	5.5.0	https://gitlab.com/ezlab/busco
bwa-mem2	2.2.1	https://github.com/bwa-mem2/bwa-mem2
Cooler	0.8.11	https://github.com/open2c/cooler
DIAMOND	2.1.8	https://github.com/bbuchfink/diamond
fasta_windows	0.2.4	https://github.com/tolkit/fasta_windows
FastK	427104ea91c78c3b8b8b49f1a7d6bbeaa869ba1c	https://github.com/thegenemyers/FASTK
Gfastats	1.3.6	https://github.com/vgl-hub/gfastats
GoaT CLI	0.2.5	https://github.com/genomehubs/goat-cli
Hifiasm	0.19.5-r587	https://github.com/chhylp123/hifiasm
HiGlass	44086069ee7d4d3f6f3f0012569789ec138f42b84aa44357826c0b6753eb28de	https://github.com/higlass/higlass
MerquryFK	d00d98157618f4e8d1a9190026b19b471055b22e	https://github.com/thegenemyers/MERQURY.FK
Minimap2	2.24-r1122	https://github.com/lh3/minimap2
MultiQC	1.14, 1.17, and 1.18	https://github.com/MultiQC/MultiQC
Nextflow	23.04.1	https://github.com/nextflow-io/nextflow
OATK	0.2	https://github.com/c-zhou/oatk
PretextView	0.2.5	https://github.com/sanger-tol/PretextView
purge_dups	1.2.3	https://github.com/dfguan/purge_dups
samtools	1.19.2	https://github.com/samtools/samtools
sanger-tol/ascc	-	https://github.com/sanger-tol/ascc
sanger-tol/blobtoolkit	0.4.0	https://github.com/sanger-tol/blobtoolkit
Seqtk	1.3	https://github.com/lh3/seqtk
Singularity	3.9.0	https://github.com/sylabs/singularity
TreeVal	1.2.0	https://github.com/sanger-tol/treeval
YaHS	1.1a.2	https://github.com/c-zhou/yahs

### Wellcome Sanger Institute – Legal and Governance

The materials that have contributed to this genome note have been supplied by a Darwin Tree of Life Partner. The submission of materials by a Darwin Tree of Life Partner is subject to the **‘Darwin Tree of Life Project Sampling Code of Practice’**, which can be found in full on the Darwin Tree of Life website here. By agreeing with and signing up to the Sampling Code of Practice, the Darwin Tree of Life Partner agrees they will meet the legal and ethical requirements and standards set out within this document in respect of all samples acquired for, and supplied to, the Darwin Tree of Life Project.

Further, the Wellcome Sanger Institute employs a process whereby due diligence is carried out proportionate to the nature of the materials themselves, and the circumstances under which they have been/are to be collected and provided for use. The purpose of this is to address and mitigate any potential legal and/or ethical implications of receipt and use of the materials as part of the research project, and to ensure that in doing so we align with best practice wherever possible. The overarching areas of consideration are:

•     Ethical review of provenance and sourcing of the material

•     Legality of collection, transfer and use (national and international)

Each transfer of samples is further undertaken according to a Research Collaboration Agreement or Material Transfer Agreement entered into by the Darwin Tree of Life Partner, Genome Research Limited (operating as the Wellcome Sanger Institute), and in some circumstances other Darwin Tree of Life collaborators.

## Data Availability

European Nucleotide Archive: *Alisma plantago-aquatica* (common water plantain). Accession number PRJEB65656; https://identifiers.org/ena.embl/PRJEB65656. The genome sequence is released openly for reuse. The *Alisma plantago-aquatica* genome sequencing initiative is part of the Darwin Tree of Life (DToL) project. All raw sequence data and the assembly have been deposited in INSDC databases. The genome will be annotated using available RNA-Seq data and presented through the Ensembl pipeline at the European Bioinformatics Institute. Raw data and assembly accession identifiers are reported in Table 1.

## Author information

Members of the Royal Botanic Gardens Kew Genome Acquisition Lab are listed here: https://doi.org/10.5281/zenodo.12625079.

Members of the Plant Genome Sizing collective are listed here: https://doi.org/10.5281/zenodo.7994306.

Members of the Darwin Tree of Life Barcoding collective are listed here: https://doi.org/10.5281/zenodo.12158331

Members of the Wellcome Sanger Institute Tree of Life Management, Samples and Laboratory team are listed here: https://doi.org/10.5281/zenodo.12162482.

Members of Wellcome Sanger Institute Scientific Operations: Sequencing Operations are listed here: https://doi.org/10.5281/zenodo.12165051.

Members of the Wellcome Sanger Institute Tree of Life Core Informatics team are listed here: https://doi.org/10.5281/zenodo.12160324.

Members of the Tree of Life Core Informatics collective are listed here: https://doi.org/10.5281/zenodo.12205391.

Members of the Darwin Tree of Life Consortium are listed here: https://doi.org/10.5281/zenodo.4783558.
