# Supplementary material for: The genome sequence of the common water plantain, Alisma plantago-aquatica L. (Alismataceae)
Source: Wellcome Open Res. 2025 Apr 23;10:209. [Version 1] doi: 10.12688/wellcomeopenres.24005.1 (PMC12125570; doi:10.12688/wellcomeopenres.24005.1)
Supplement: Peer review history

# Reviewer report: Reviewer response for version 1

**DOI:** 10.21956/wellcomeopenres.26483.r122622

**Published:** 2025-05-30

**Authors:** Angela McDonnell

**Affiliations:** 1 St Cloud State University, Saint Cloud, Minnesota, USA

The manuscript reports on the sequencing, assembly, and statistics concerning the assembly of Alisma plantago-aquatica, which is the first species to be sequenced at this scale for the family. The assembly appears to corroborate previous reports of ploidy and alludes to the utility of the new sequence in the elucidation of biochemical pathways important in medicine. The only area that I can detect would be improved by more detail would the the utility of the sequences obtained and the motivation for the work; I find the comments in these areas to be sparse. 

 Despite that, I find the work to be concise, clear, complete, and contain all absolutely necessary components of a new genome report Indexed. The work is scientifically sound as is. I do think the addition of settings and code used to run the software/packages used would strengthen the data availability/open access nature of the work. These could be placed in github, for example.

Are sufficient details of methods and materials provided to allow replication by others?

Yes

Is the rationale for creating the dataset(s) clearly described?

Yes

Are the datasets clearly presented in a useable and accessible format?

Yes

Are the protocols appropriate and is the work technically sound?

Yes

Reviewer Expertise:

Phylogenomics, systematics, taxonomy

I confirm that I have read this submission and believe that I have an appropriate level of expertise to confirm that it is of an acceptable scientific standard.

# Reviewer report: Reviewer response for version 1

**DOI:** 10.21956/wellcomeopenres.26483.r123565

**Published:** 2025-05-28

**Authors:** Thomas J. Givnish

**Affiliations:** 1 University of Wisconsin-Madison, Madison, USA

This is a straightforward report on obtaining a whole-genome sequence of Alisma plantago-aquatica. The account is well organized and clearly written. I had only three suggestions:

 1. The ms states that this is the first WGS for Alismataceae, which is correct. Perhaps the authors might to note that several families of order Alismatales are now represented in GenBank (e.g., Araceae, Juncaginaceae, Zosteraceae) and that these will jointly contribute to our understanding of gene evolution in a group of mostly wetland and aquatic species. It wasn't clearly to me whether several recent or in-progress genomes at GenBank from the Wellcome Sanger Institute involved the same or different authors. Perhaps the authors might mention those efforts as well.

 2. While the assembly number is given in Table 2, nowhere in the text is the fact that it is (or is about to be) deposted in GenBank.

 3. There is a discrepancy between the sequenced length (ca. 9378 Mb, with nearly 250 gaps) and the flow cytometry estimate of genome size (ca. 10,580 Mb). Discrepancies of this size (in this case, 12.8%) often arise. But I do wonder whether a systematic error might be in play. See the unreviewed preprint by Soni & Henry in BioRchiv: https://www.biorxiv.org/content/10.1101/2024.11.11.623134v2 . Not sure whether the authors will want to mention this potential factor inflating cytometry estimates of genomes size.

Are sufficient details of methods and materials provided to allow replication by others?

Yes

Is the rationale for creating the dataset(s) clearly described?

Yes

Are the datasets clearly presented in a useable and accessible format?

Yes

Are the protocols appropriate and is the work technically sound?

Yes

Reviewer Expertise:

Plant ecology, molecular phylogenetics and phylogenomics, historical biogeography

I confirm that I have read this submission and believe that I have an appropriate level of expertise to confirm that it is of an acceptable scientific standard.
